# Supplementary material for: Hidden pressurized fluids prior to the 2014 phreatic eruption at Mt Ontake
Source: Nat Commun. 2022 Oct 17;13:6145. doi: 10.1038/s41467-022-32252-w (PMC9576684; doi:10.1038/s41467-022-32252-w)
Supplement: Supplementary file 1 — Supplementary information [file 41467_2022_32252_MOESM1_ESM.pdf]

**Title**

- Hidden pressurized fluids prior to the 2014 phreatic eruption at Mt Ontake

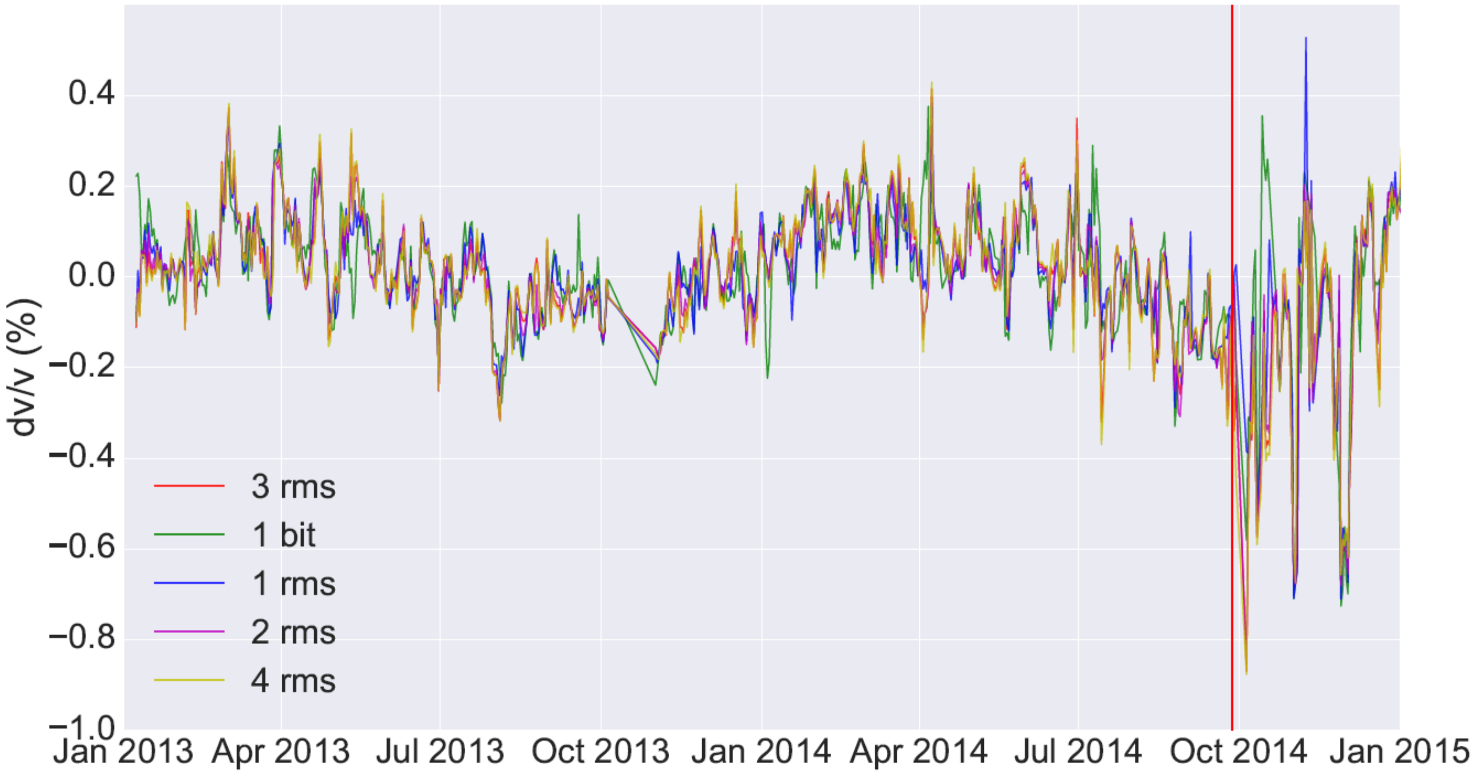

Caption Supplementary Fig.1. **dv/v -rms**: Relative velocity variations are estimated by performing 1-bit normalization and clipping the autocorrelation functions (station ONTA) with different RMS (Root Mean Squares) values. The red line indicates the 27 September 2014 eruption. The dv/v are very similar.

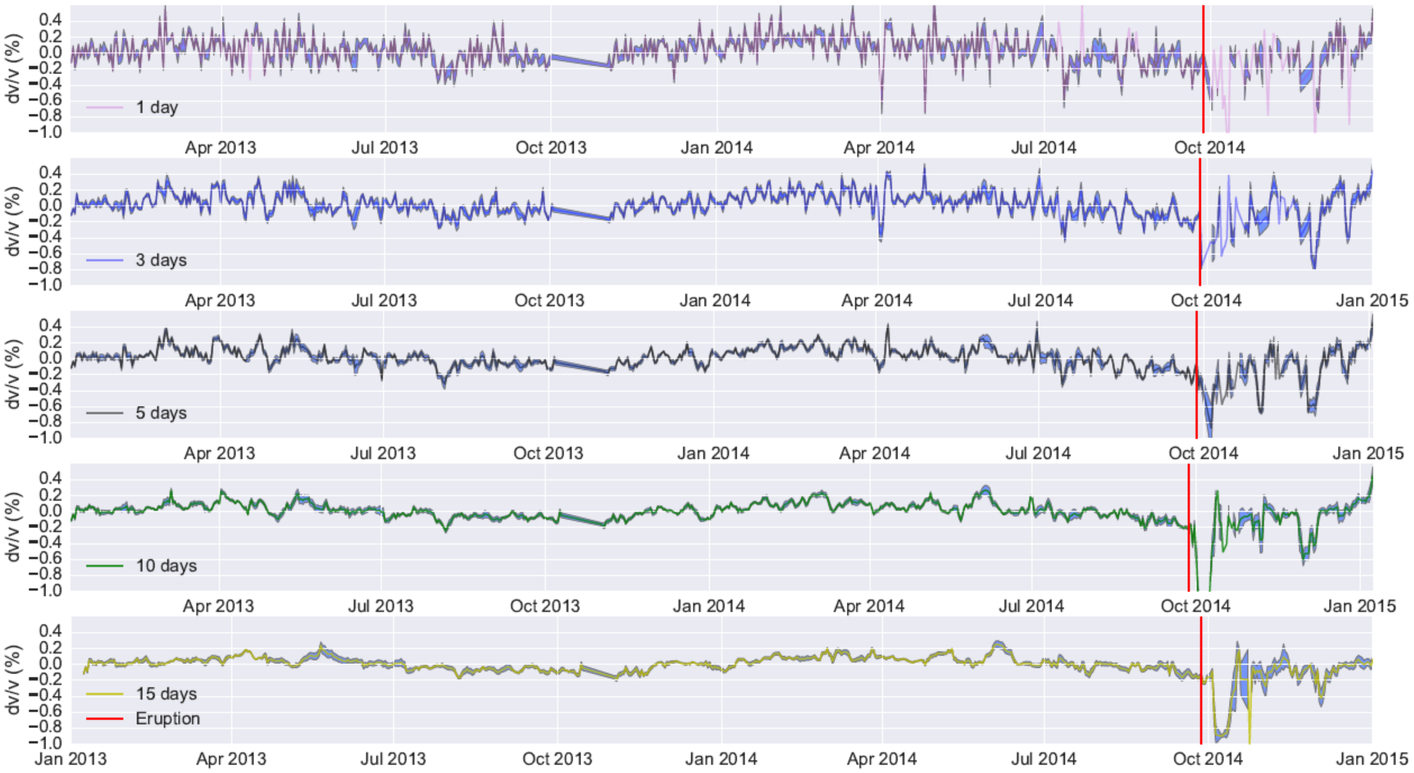


Caption Supplementary Fig.2. **dv/v – stacking**. Relative velocity variations are estimated by stacking (linear stack) different number of days of autocorrelation functions (station ONTA). The red line indicates the 27 September 2014 eruption. Errors are shown as blue shaded values around each curve and are estimated following Lecocq et al.[1]. The 1-day dv/v were noisy with too large errors (no errors shown around the line a few days after the eruption).

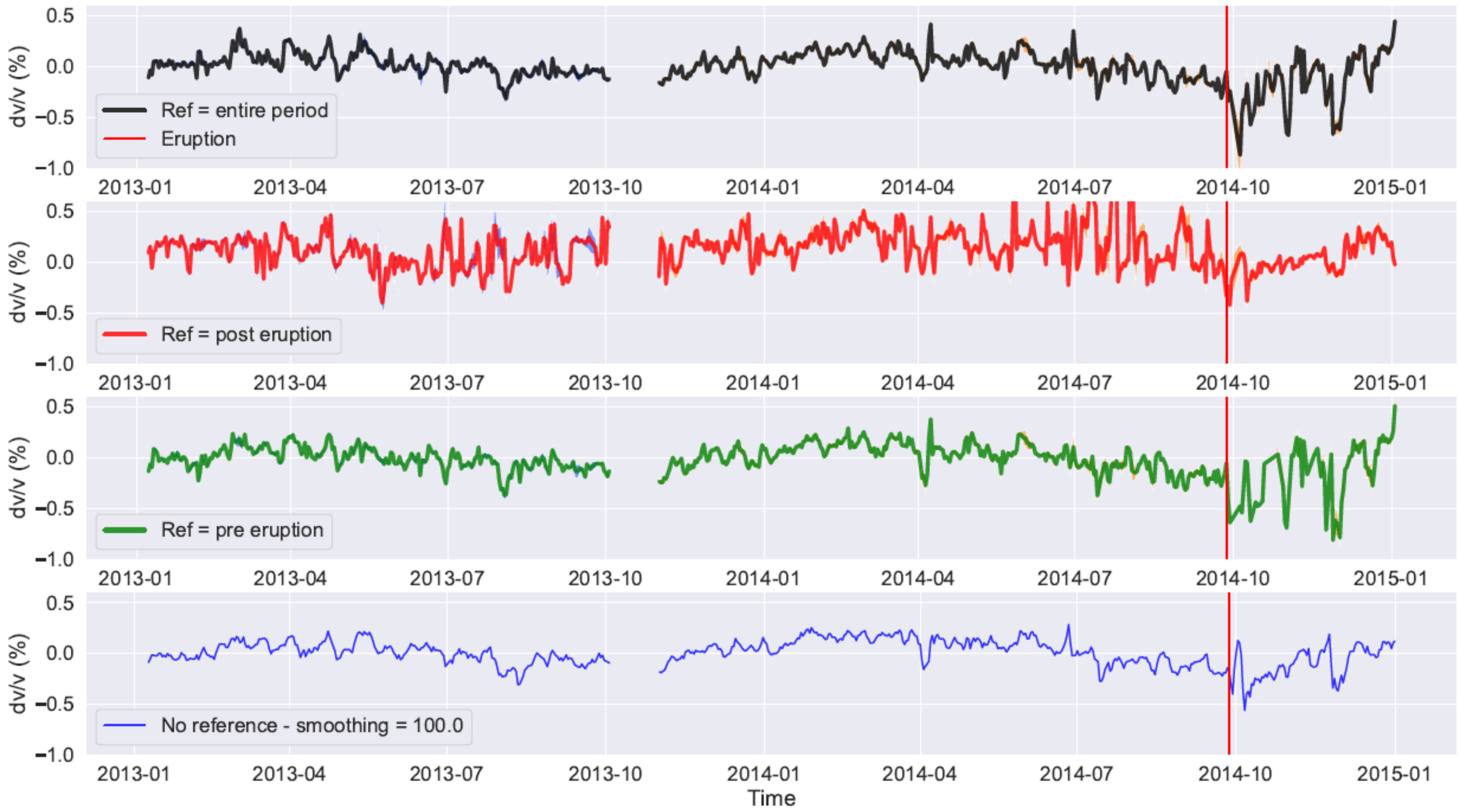


Caption Supplementary Fig.3. **dv/v-ref**. Relative velocity variations are estimated by comparing the 5-day stacks with a reference computed using different time periods. The red line indicates the 27 September 2014 eruption. Errors are shown as blue shaded values around

each curve and are estimated following Lecocq et al.[1]. The post-eruption period is not coherent enough to compute reliable dv/v estimates, as observed in Supplementary Fig.5.

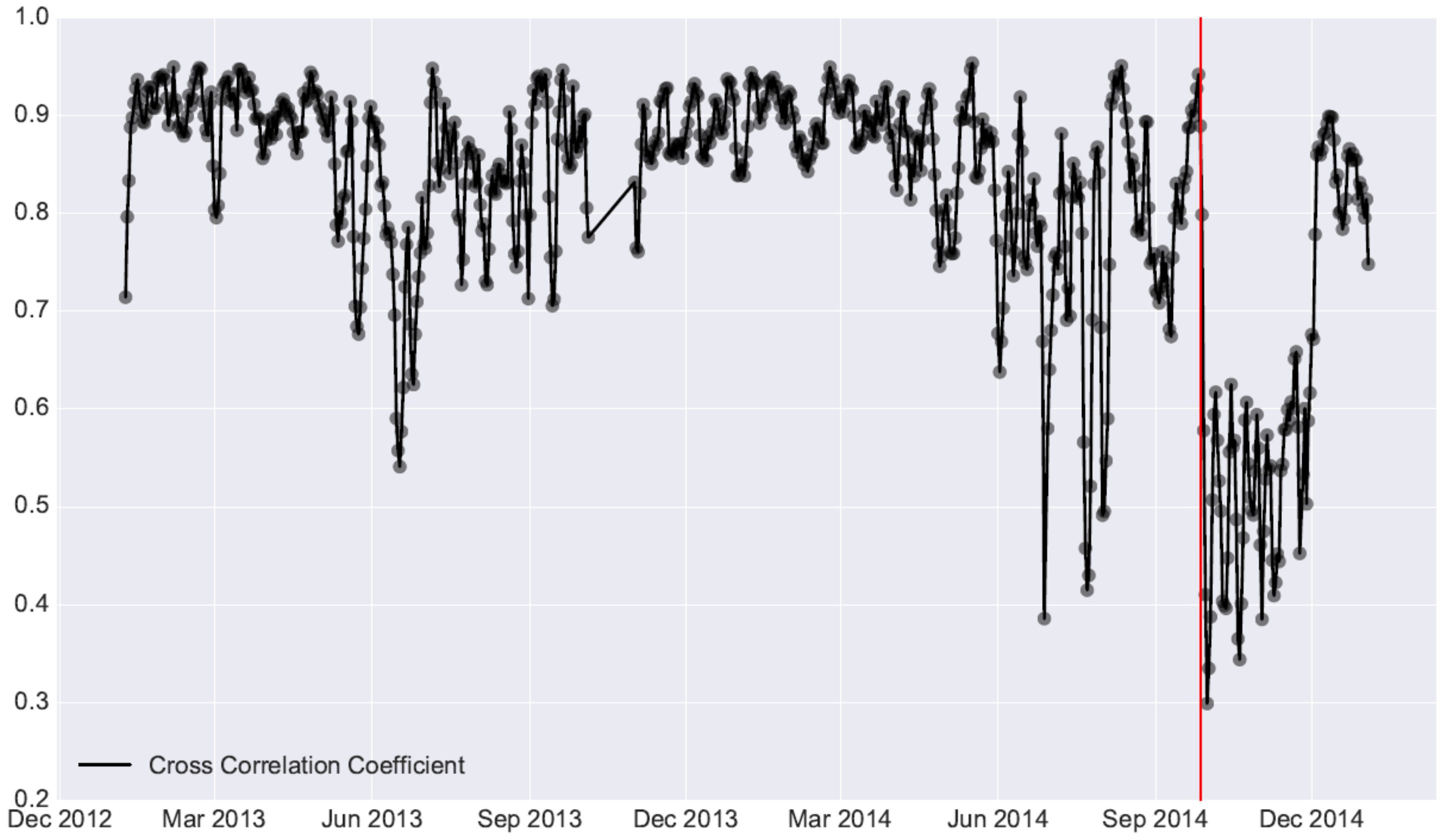

Caption Supplementary Fig.4. **dv/v-CCC**. The cross correlation coefficient between the reference of the daily AC functions (between +-5 to +-35 s) are generally above 0.7 (Supplementary Fig.5) except after the eruption until December 2014. Pre-eruptive values were only recovered in December and could indicate that the medium was not extensively disrupted by the eruption.

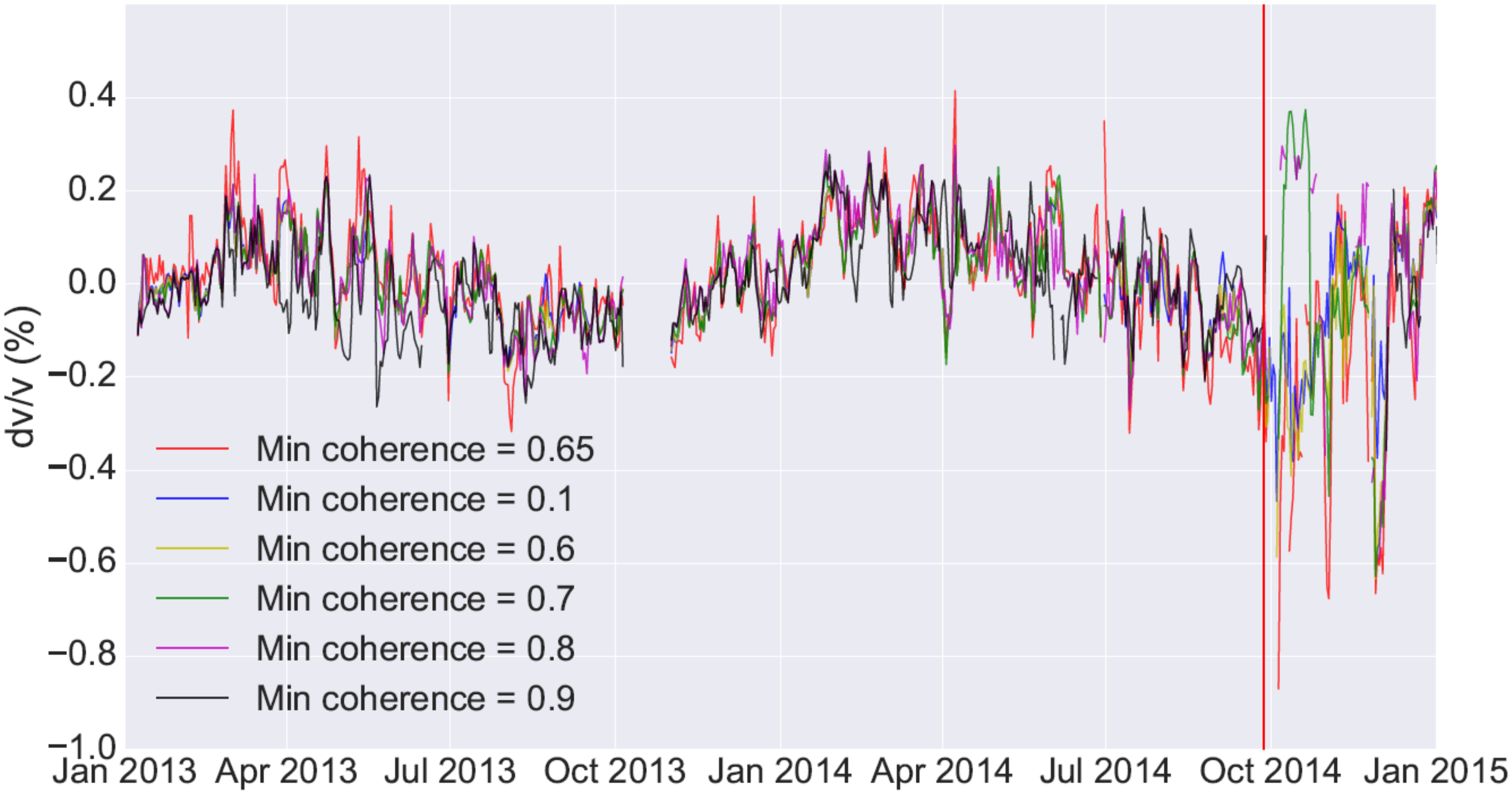

Caption Supplementary Fig.5. **dv/v-coherence**. Relative velocity variations are estimated based on different thresholds for the minimal value of coherence, as defined by Lecocq et al. [1]. The red line indicates the 27 September 2014 eruption. Results are similar except after the eruption, between October and December 2014, when low values of coherence were observed. As a result, the dv/v were only computed for low values of coherences; black and magenta curves almost disappear between October and November 2014.

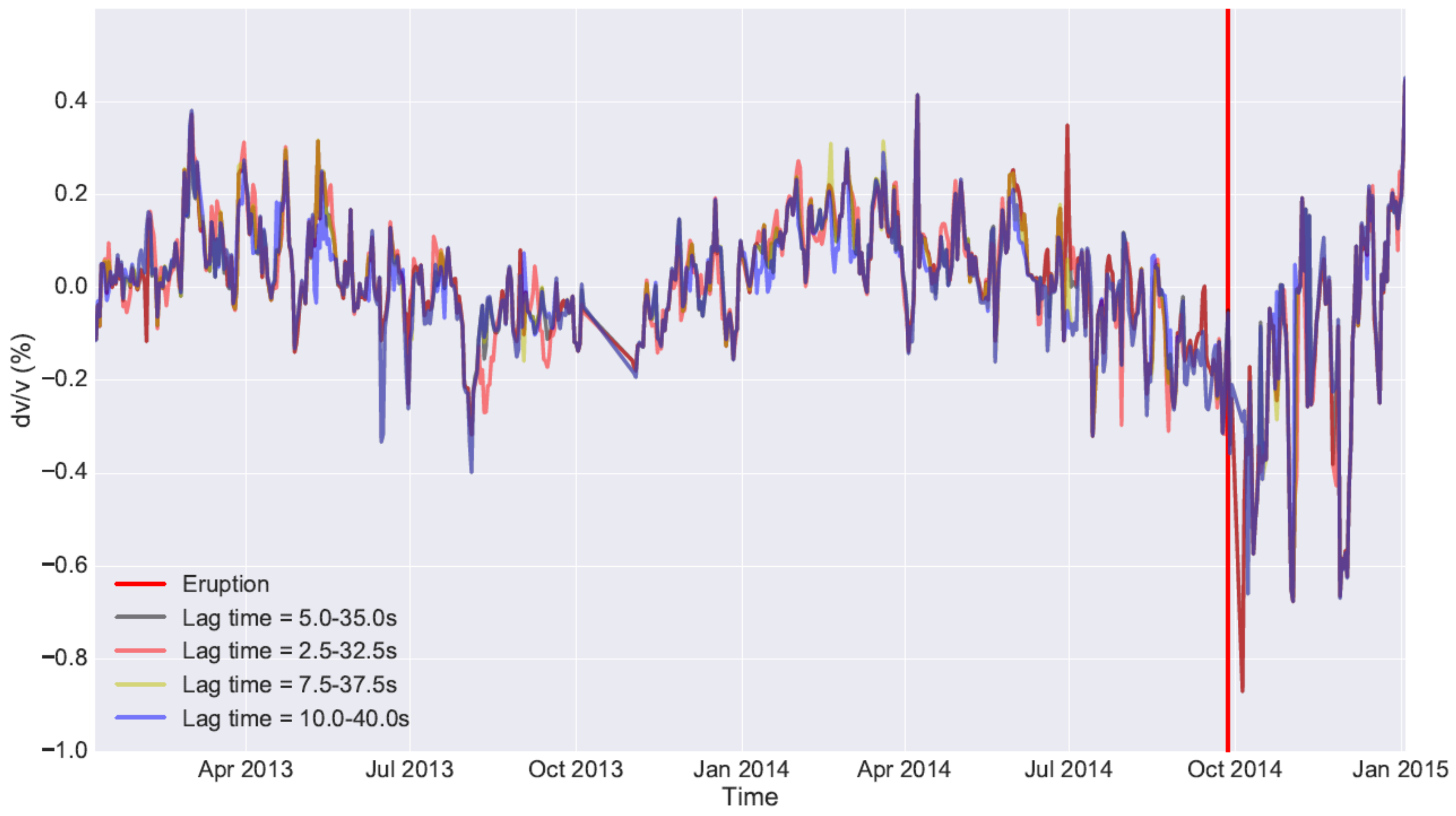


Caption Supplementary Fig.6. **dv/v-lag time**. Relative velocity variations are estimated using auto correlations at different lag times using station ONTA. The red line indicates the 27 September 2014 eruption. The results are overall similar.

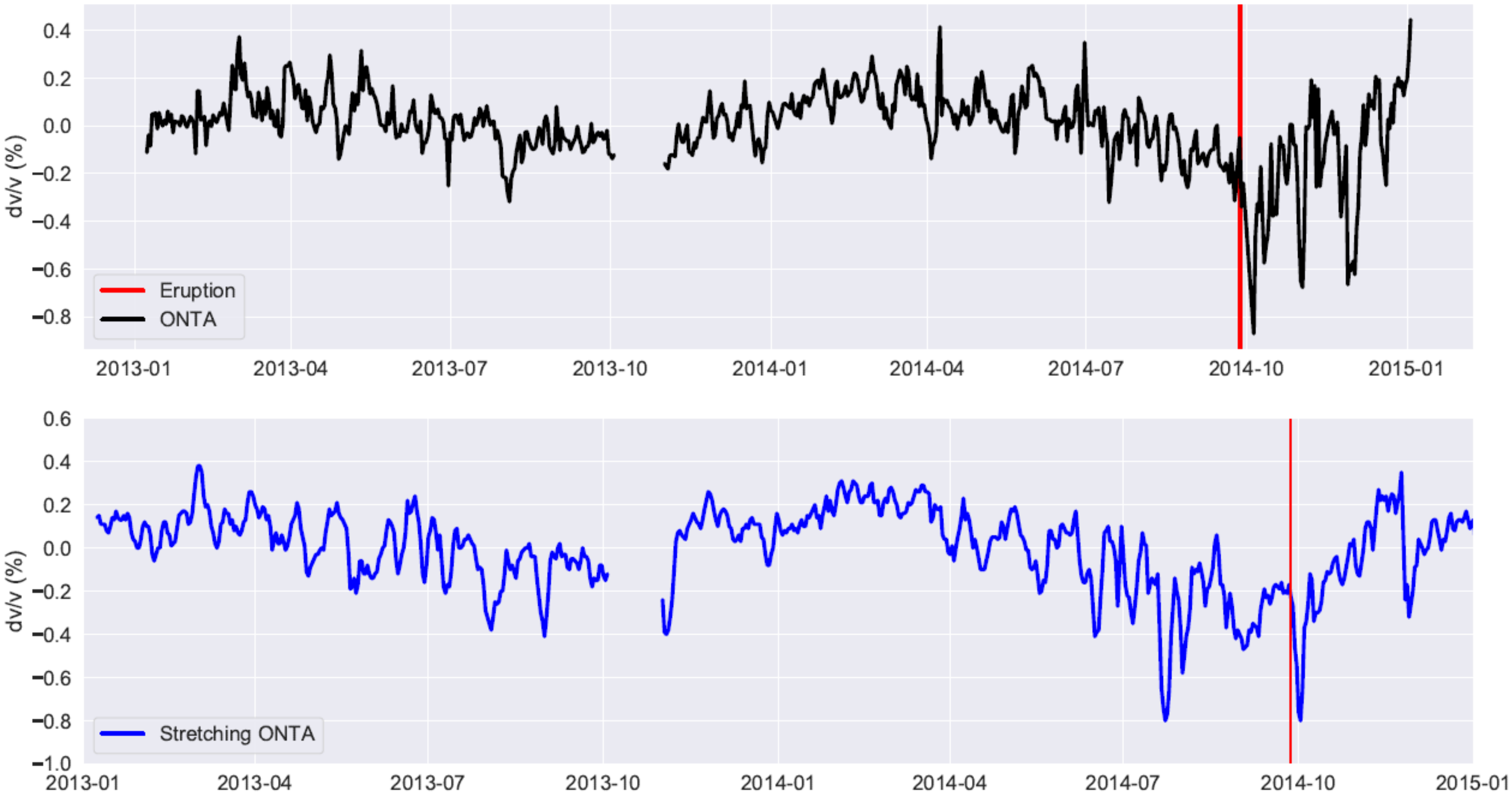


Caption Supplementary Fig.7. **dv/v-stretching**. Comparison between the Moving Window Cross Spectral (top panel, black line) approach and the stretching method (bottom panel, blue line, e.g., Sens-Schonfelder and Wegler[2]) to estimate the relative velocity variations. Seasonal variations appear more pronounced with the stretching method, as suggested by Zhan et al. [42]. Overall dv/v estimates are very similar and the 3 months pre-eruptive drop is also highlighted.

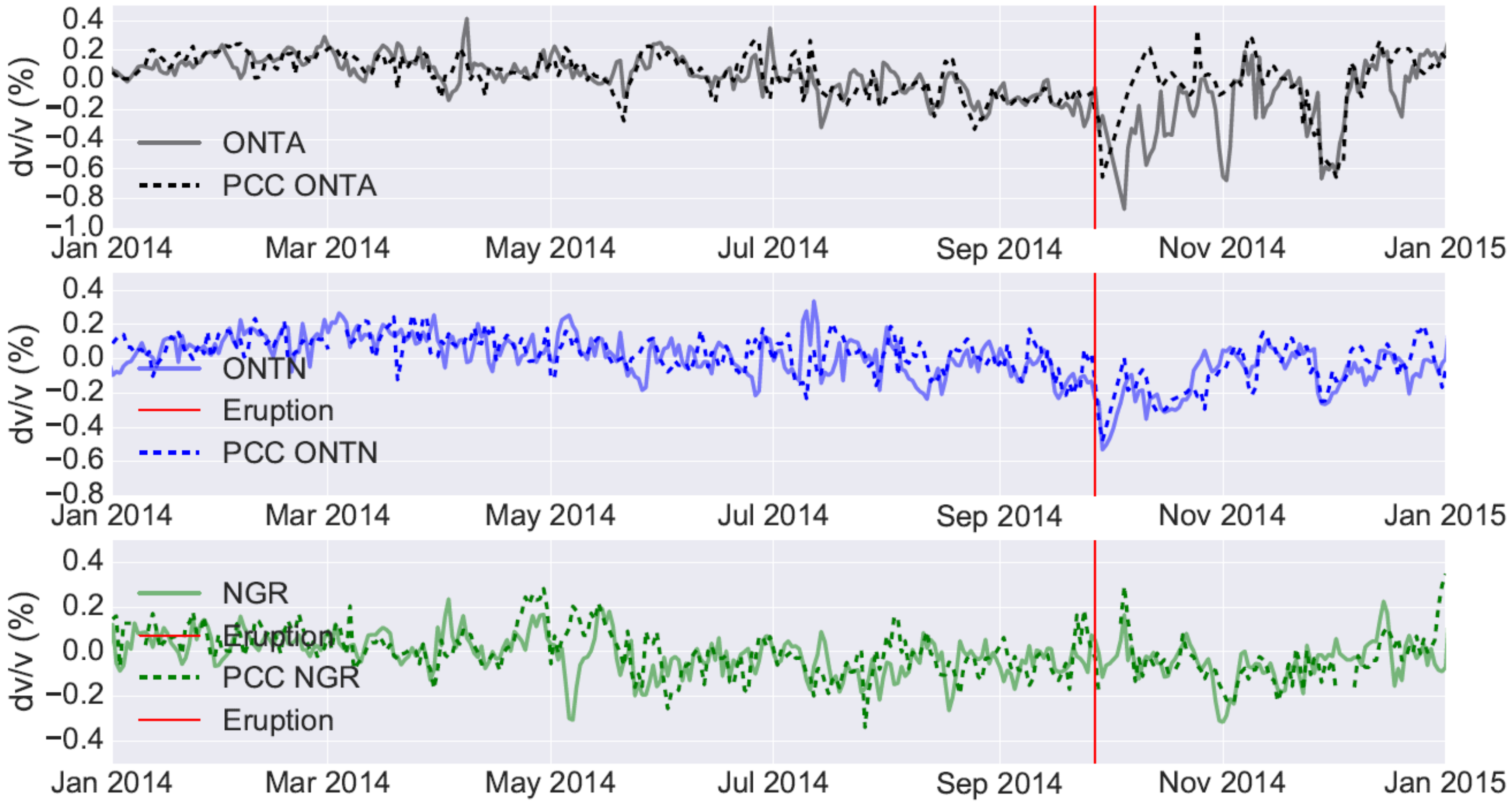


Caption Supplementary Fig.8. **dv/v - PCC**. Relative velocity variations are estimated using classical auto correlations (Lecocq et al. [1]) and the Phase Auto Correlation (dashed lines) derived from Schimmel et al.[3]. The red line indicates the 27 September 2014 eruption.

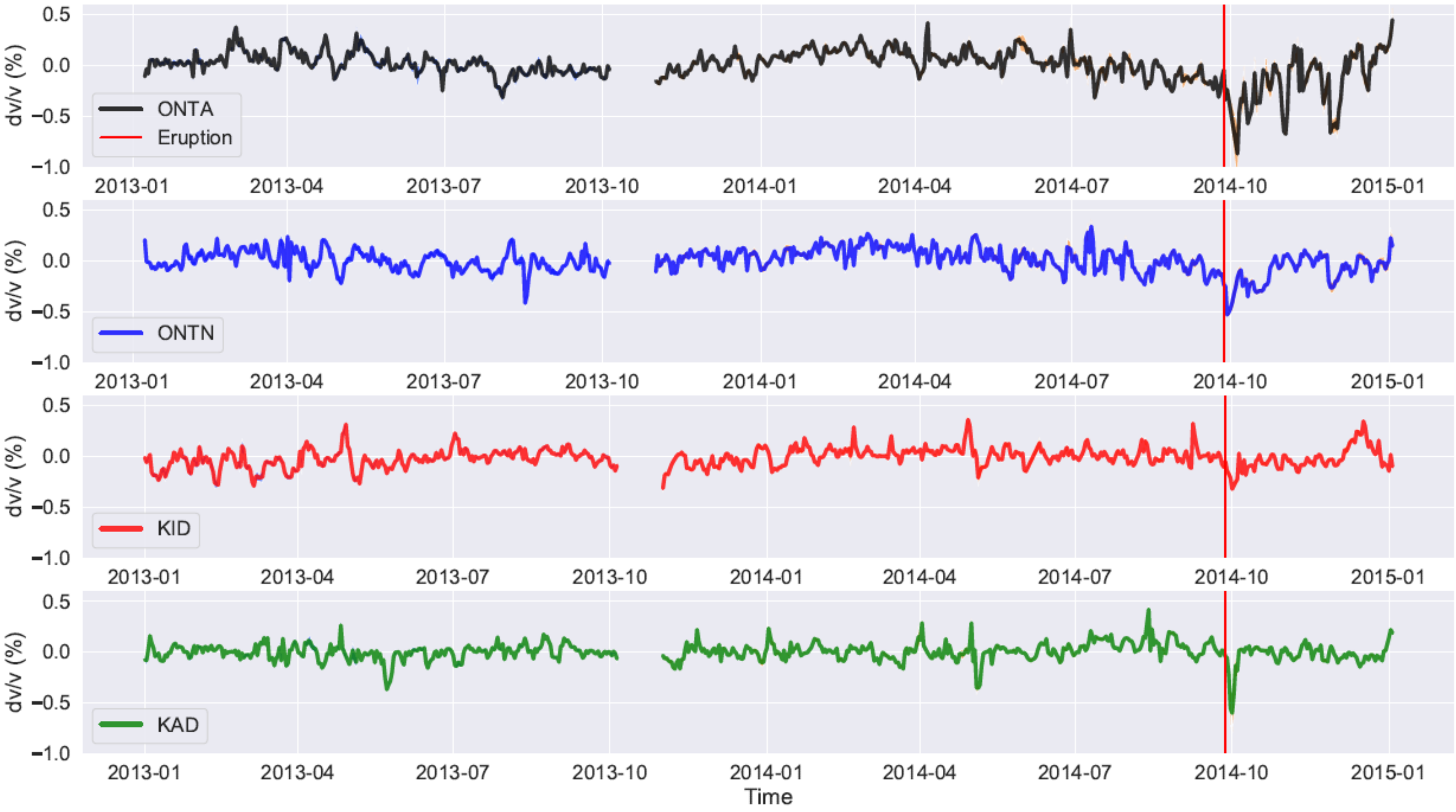


Caption Supplementary Fig.9. **dv/v-eastern flank**. Relative velocity variations for stations located on the eastern flank. The red line indicates the 27 September 2014 eruption. Errors are shown as blue shaded values around each curve and are estimated following Lecocq et al.[3].

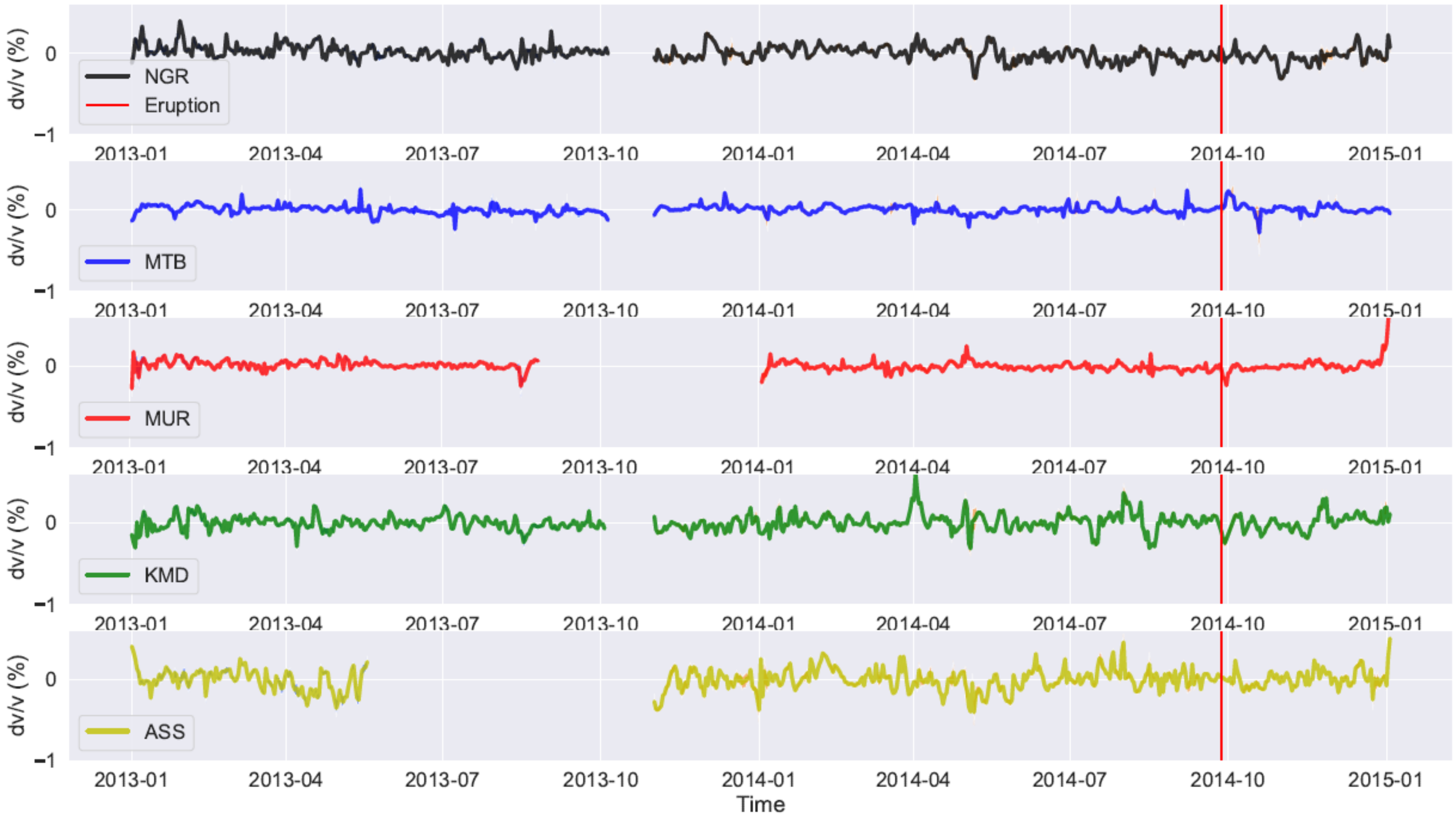


Caption Supplementary Fig.10. **dv/v – other stations**. Relative velocity variations for the other stations of the network. The red line indicates the 27 September 2014 eruption. Errors are shown as red shaded values around each curve and are estimated following Lecocq et al.[3].

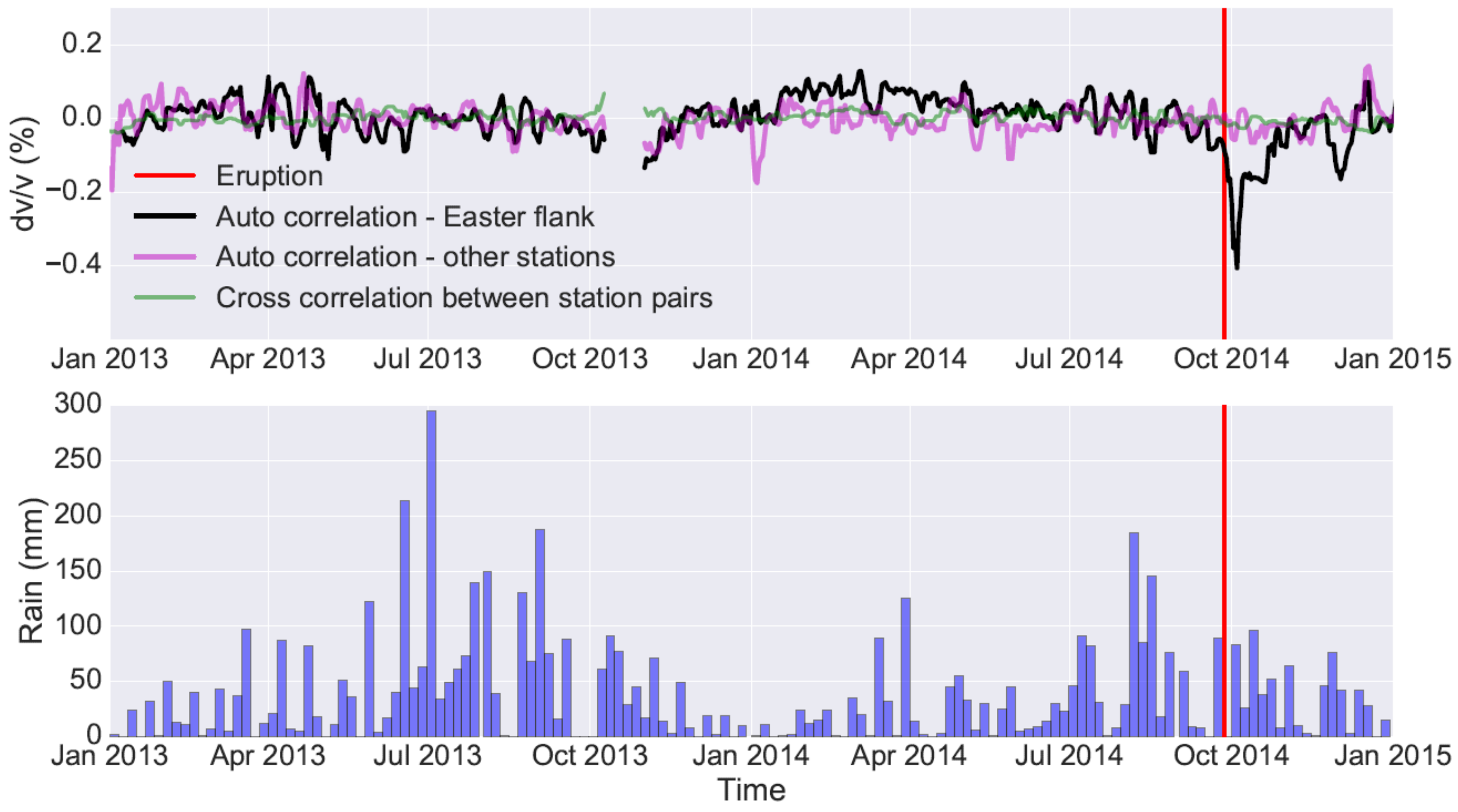


Caption Supplementary Fig.11. **dv/v – rain**. Relative velocity variations from AC-CC and meteorological data. Upper: dv/v are computed using auto correlations (black and magenta curves) and cross correlations (green curve). 5-day stacks with the full period as the reference function are used to estimate the dv/v using the Moving Window Cross Spectral Analysis (MWCS). The red line indicates the 27 September 2014 eruption. Lower: rain data from the station co-located with ONTA (Figure 1).

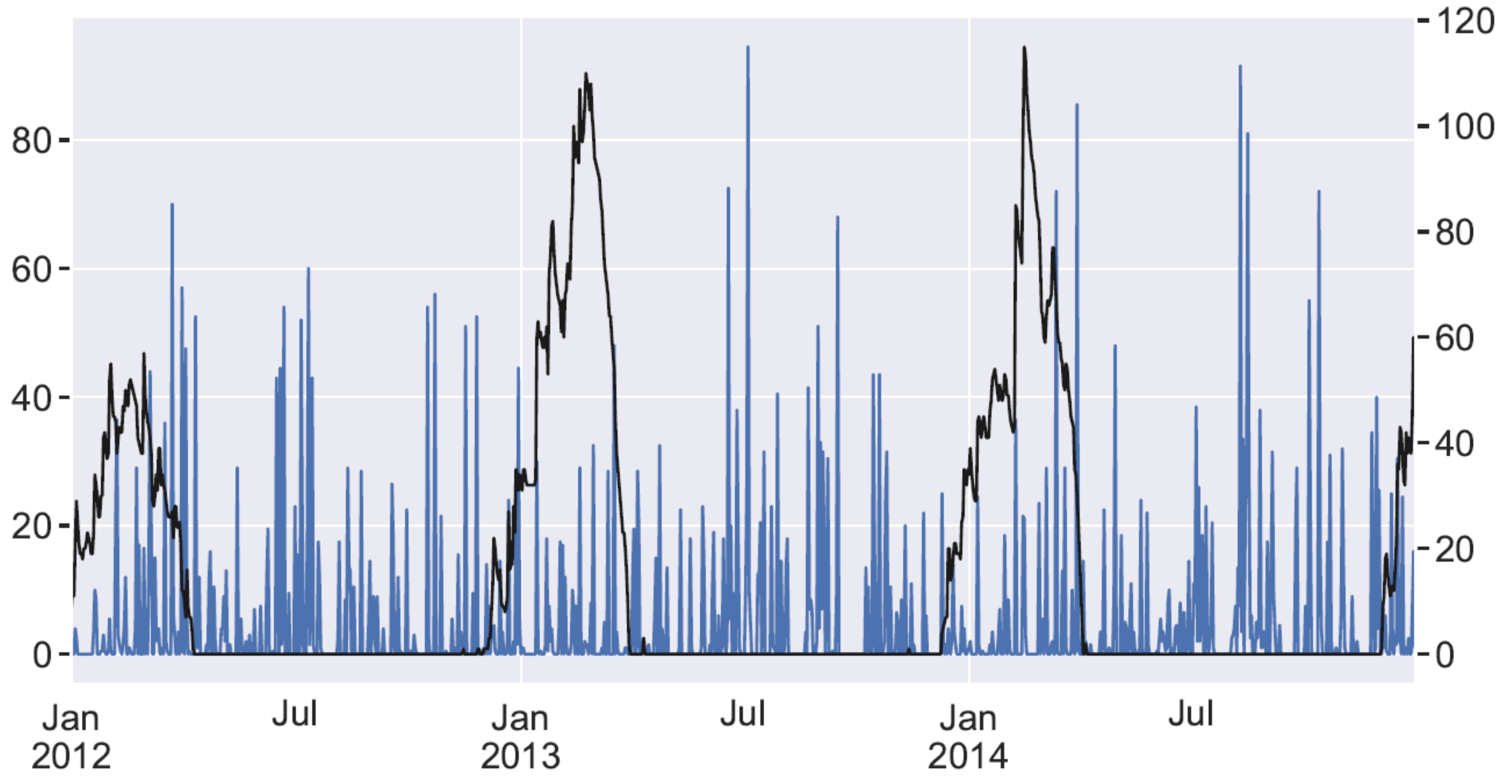


Caption Supplementary Fig.12. **dv/v – snow**. Rain (blue line) and snow (black) measured at the station co-located with ONTA (Figure 3).

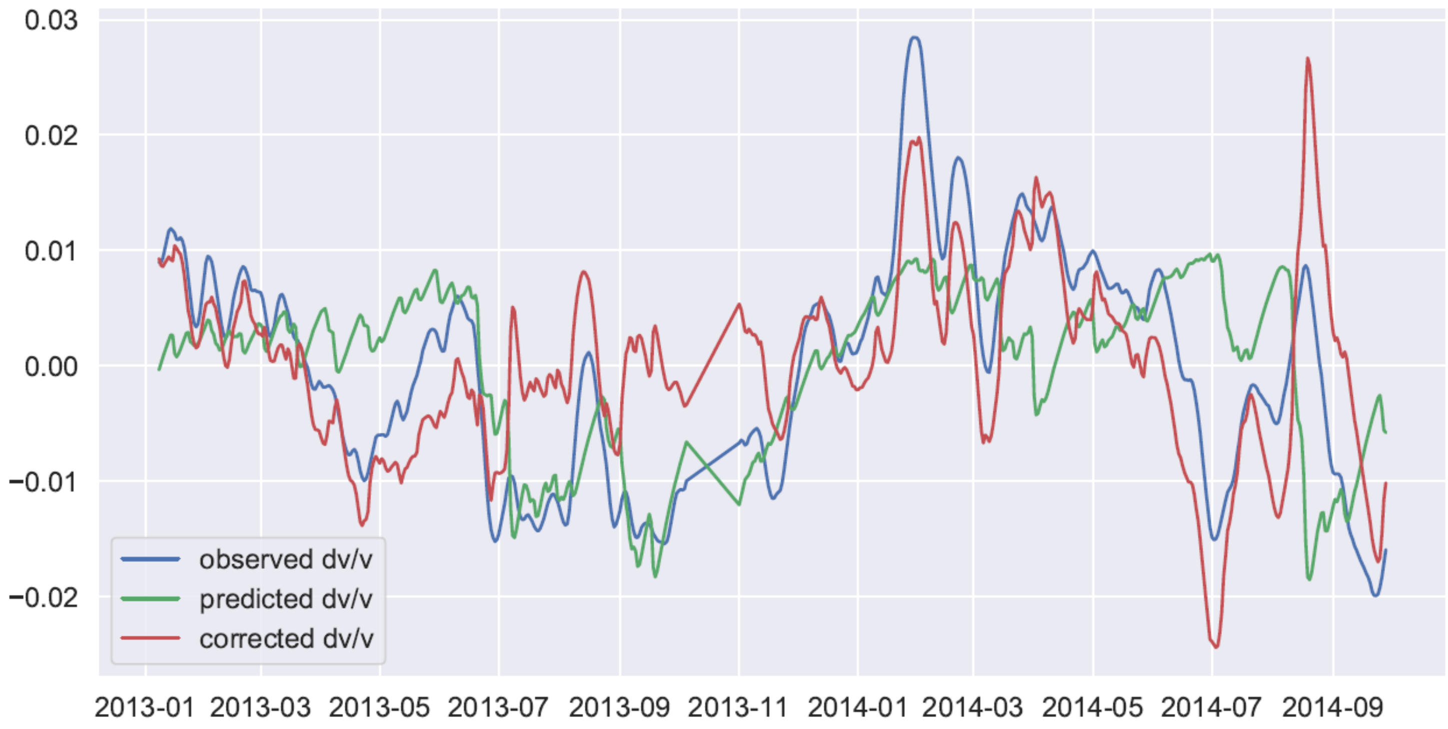


Caption Supplementary Fig.13. **dv/v – modeling**. Time evolution of the observed (blue line), the predicted dv/v (green line) and their difference (red line). dv/v values are in percent.

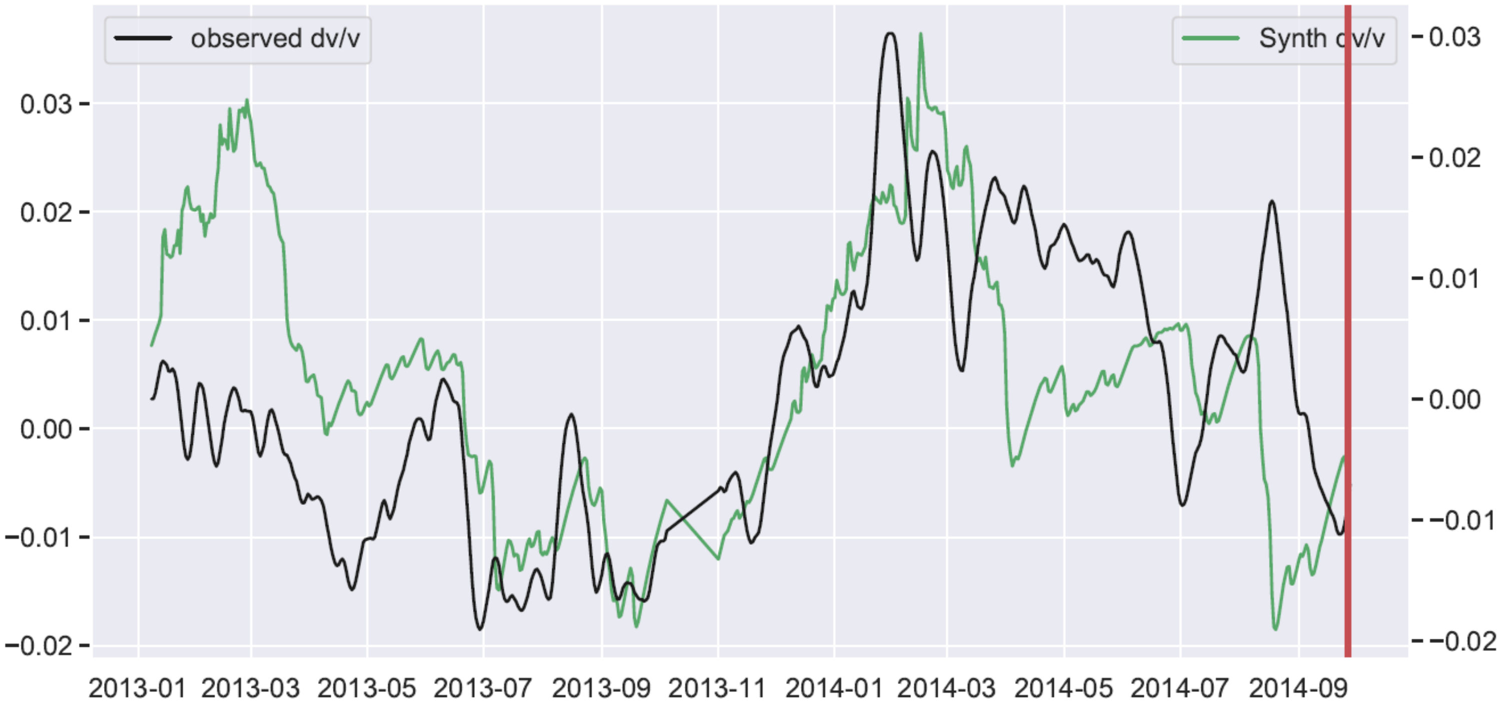


Caption Supplementary Fig.14. **dv/v – modeling synthetic**. Time evolution of the synthetic and observed dv/v. The red line indicates the 27 September 2014 eruption. dv/v values are in percent.

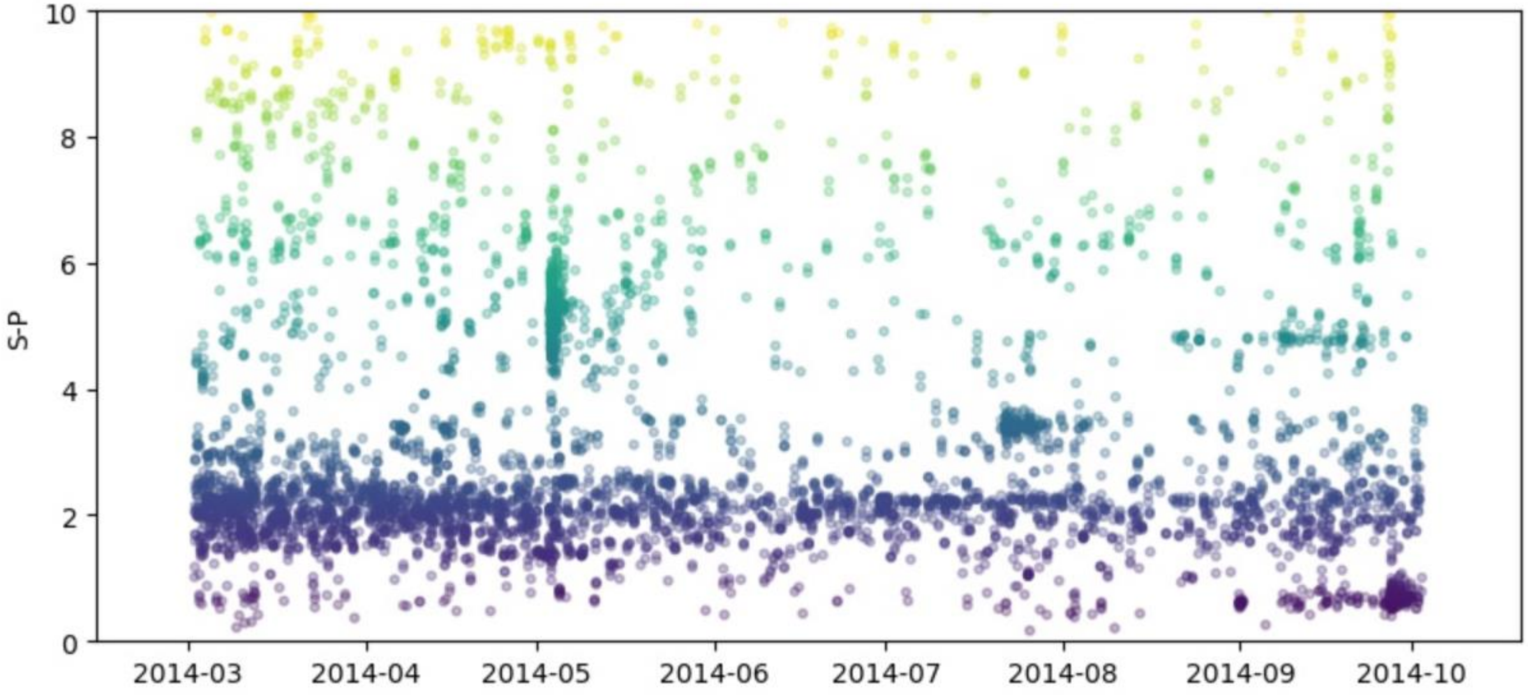


Caption Supplementary Fig.15. **Deep learning results**. Difference between P and S wave arrivals picked by the EQTransformer deep-learning model[4].

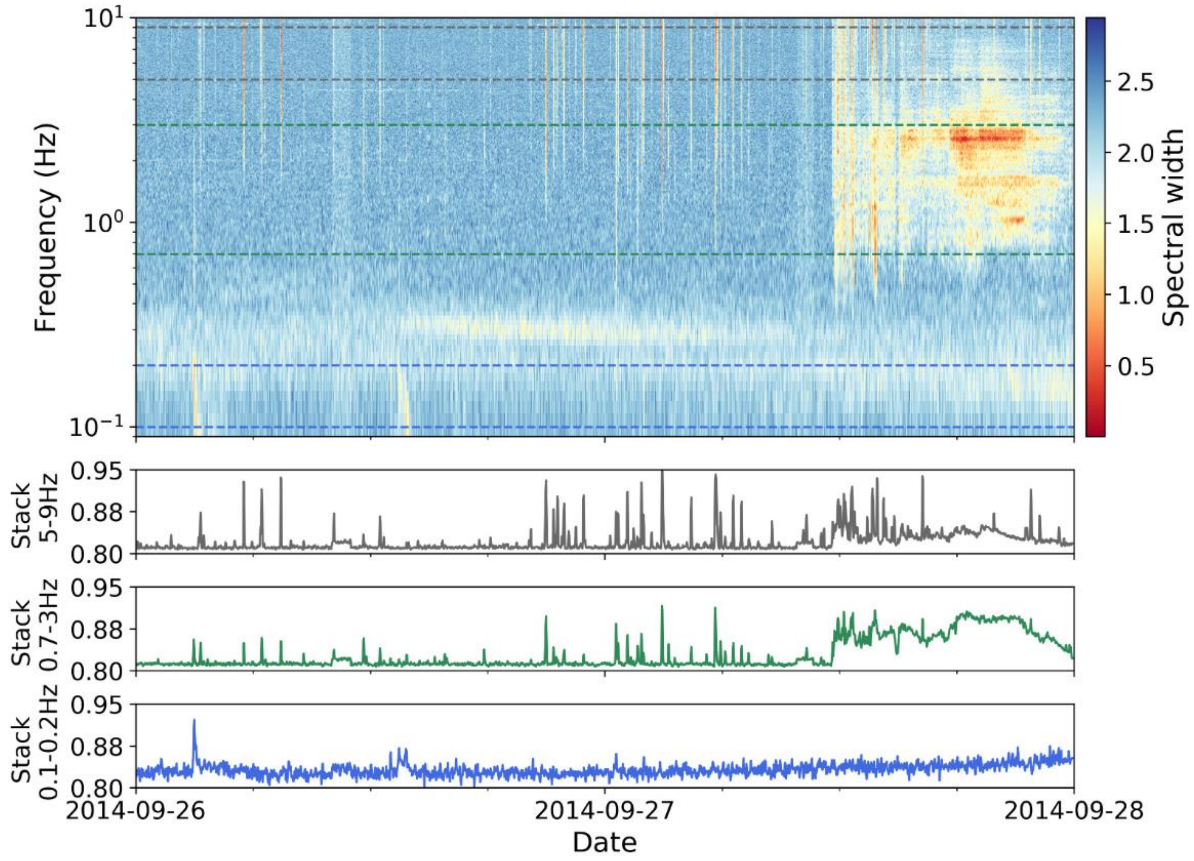


Caption Supplementary Fig.16. **Eruptive tremor**. Eruptive tremor detected by the network-based approach between 1 and 4 Hz. The spectral width of the network covariance matrix eigenvalues distribution is a proxy of the number of independent seismic sources composing the wavefield. A low value indicates a coherent signal in the network. The *stack* value in bottom panels corresponds to the normalized spectral width values in the frequency band of interest (1 - *stack* / $N_{sta}$ x $N_f$), where $N_{sta}$ is the number of stations and $N_f$ the size of the frequency band).

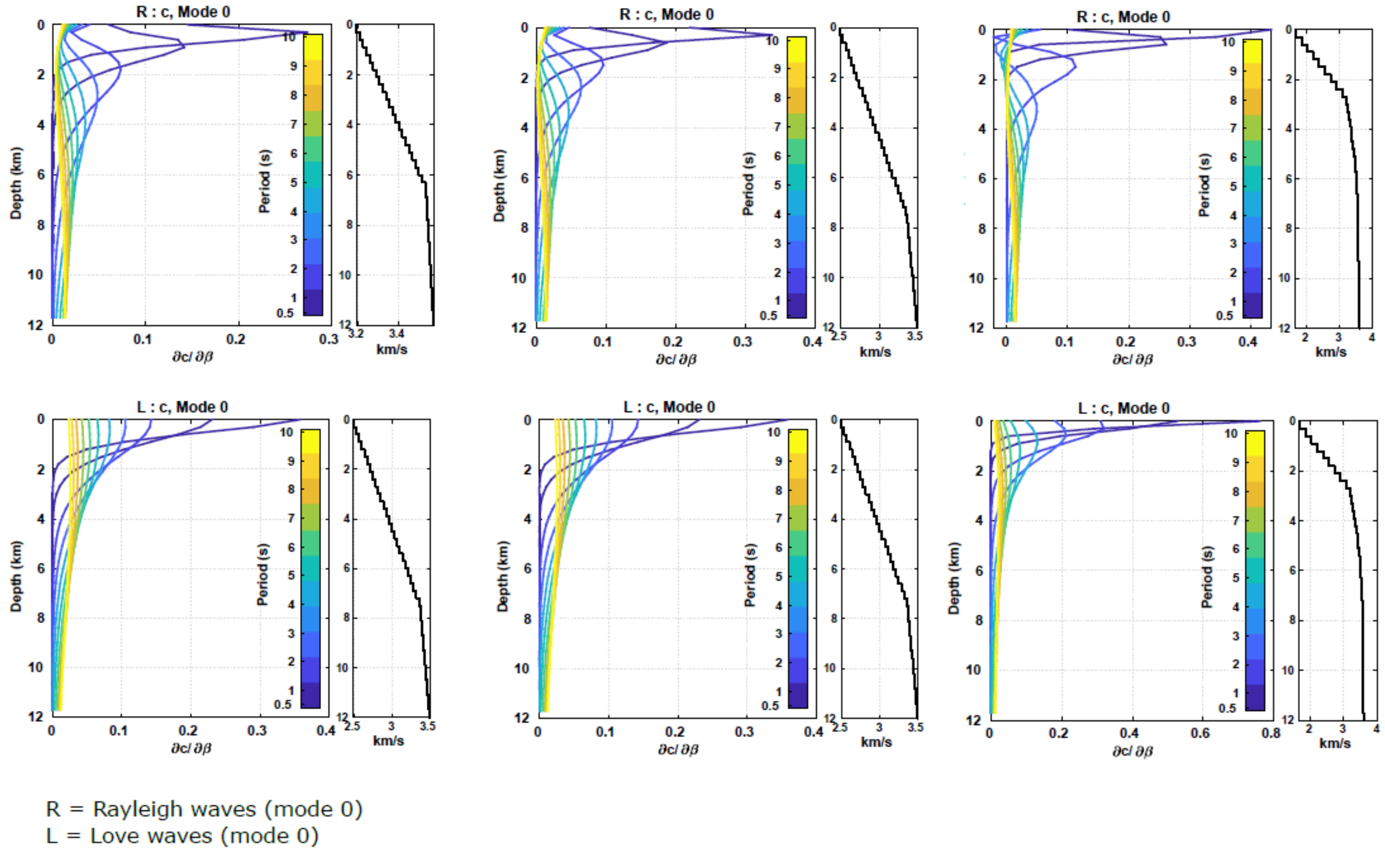


Caption Supplementary Fig.17. **Sensitivity kernels**. The phase-velocity sensitivity kernels computed for Rayleigh and Love waves using using 1D velocity models and the surf96 program[5]. We tested the 3 models shown in Terakawa et al.[6].

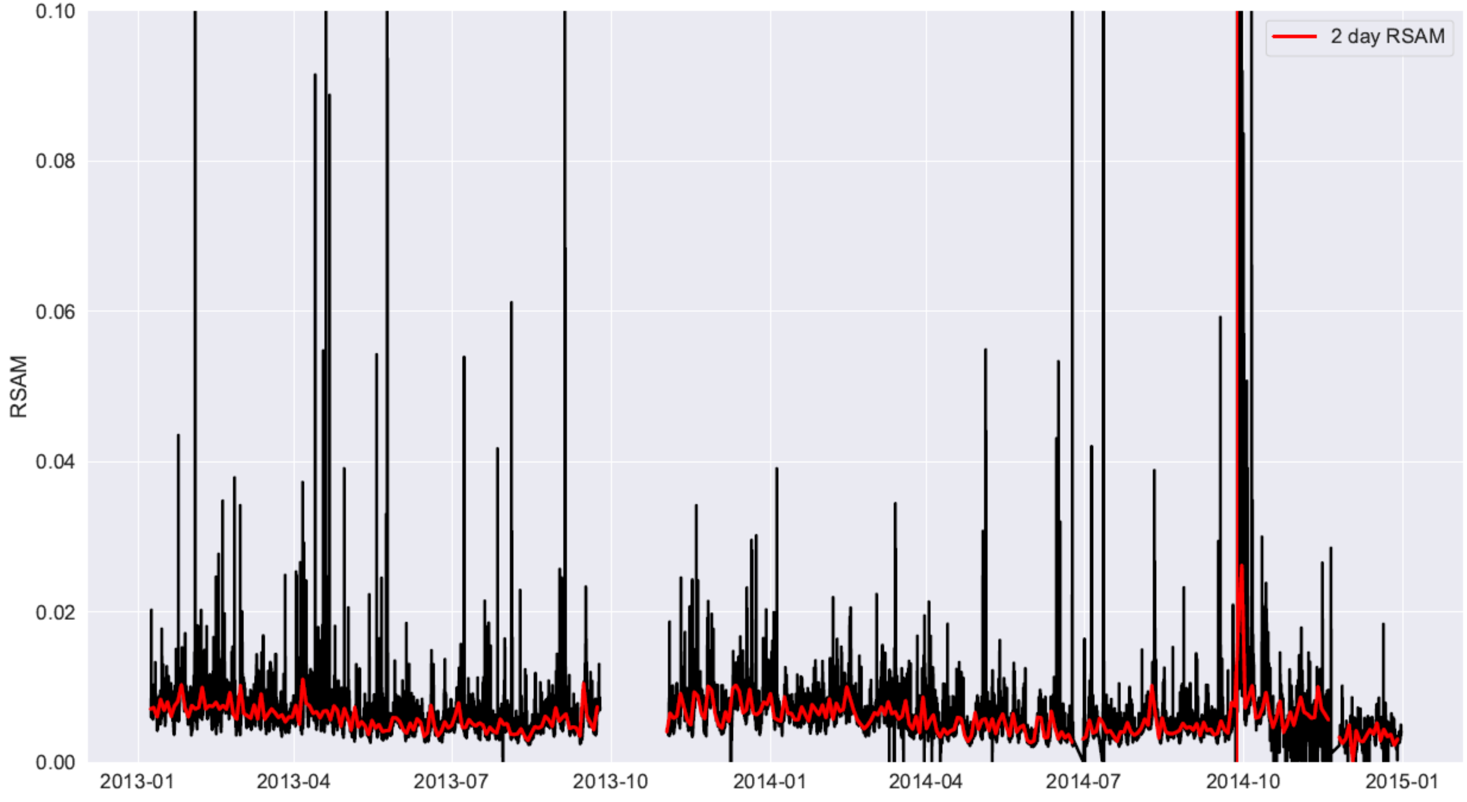


Caption Supplementary Fig.18. **RSAM**. RSAM (i.e., time evolution of the seismic amplitude) computed using seismic data from station ONTA. The 10-min resolution data (black curve) have been resampled to 2 day (red curve).

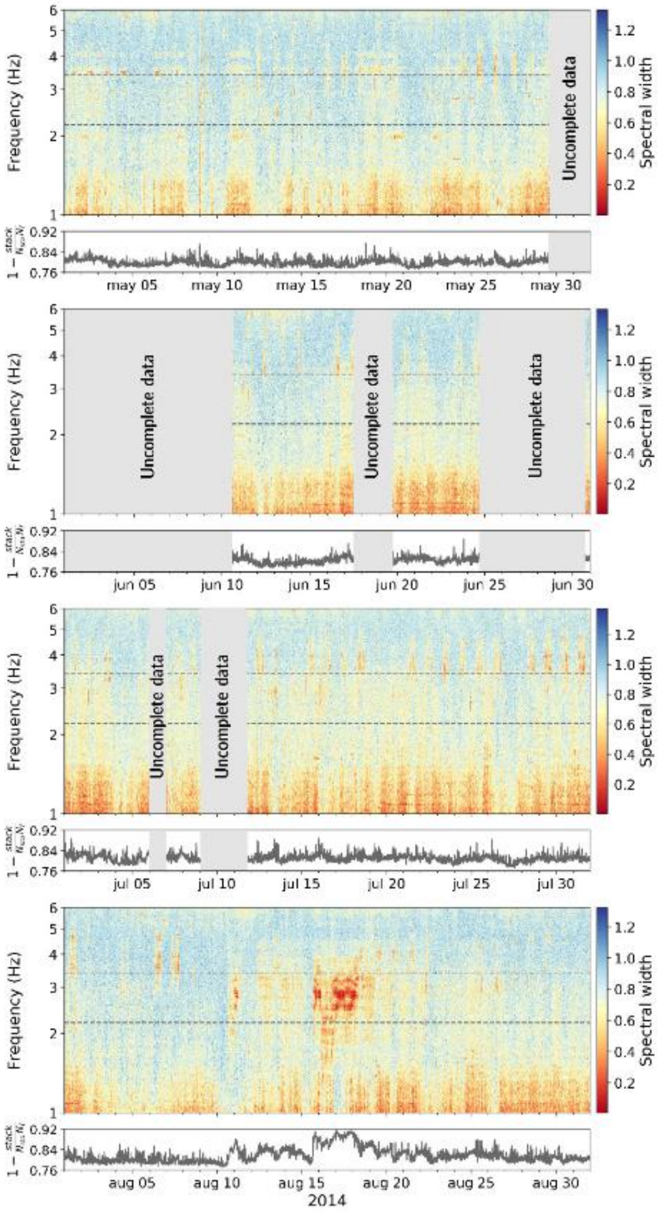


Caption Supplementary Fig.19. **Covseisnet results May to August**. Results using the network-based approach between 1 and 4 Hz for the period ranging from May to August 2014. The spectral width of the network covariance matrix eigenvalues distribution is a proxy of the number of independent seismic sources composing the wavefield. A low value indicates a coherent signal in the network. The *stack* value in bottom panels corresponds to the normalized spectral width values in the frequency band of interest (1 - *stack* / $N_{sta}$ x $N_f$), where $N_{sta}$ is the number of stations and $N_f$ the size of the frequency band).

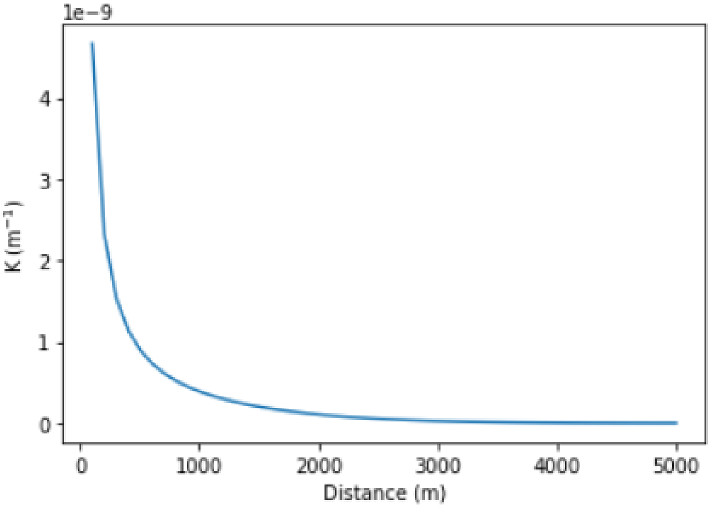


Caption Supplementary Fig.20. **Sensitivity kernel diffusion**. Sensitivity kernel (K) as a function of distance for travel-time t = 20s, D = c*l/3, where D is the diffusion coefficient, c = 1 km/s is the velocity and l=1000 m the mean free path (Pacheco and Snieder[7]).

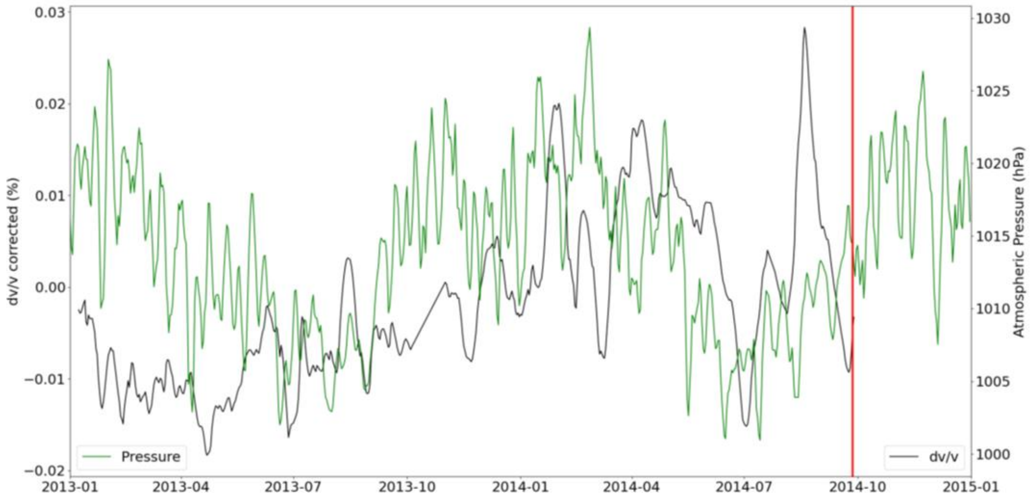


Caption Supplementary Fig.21. **dv/v – Atmospheric pressure**. Time evolution of the observed dv/v (black, in percent) and the daily atmospheric pressure (recorded in Matsumoto (36.246N 137.92E)). The red line indicates the 27 September 2014 eruption. dv/v values are in percent.

| | | |
|---|---|---|
| analysis_duration | Duration of the Analysis (total in seconds : 3600, [86400]) | 86400.0 |
| resampling_method | Resampling method | Lanczos |
| preprocess_lowpass | Preprocessing Low-pass value in Hz | 8.0 |
| preprocess_highpass | Preprocessing High-pass value in Hz | 0.0 |
| maxlag | Maximum lag (in seconds) | 120.0 |
| corr_duration | Data windows to correlate (in seconds) [1800.] | 1800.0 |
| overlap | Amount of overlap between data windows [0:1] | 0.0 |
| windsorizing | Windsorizing at N time RMS | 3.0 |
| ref_begin | Beginning or REF stacks | 41275.0 |
| ref_end | End or REF stacks | 42005.0 |
| mov_stack | Number of days to stack for the Moving-window stacks ([5]= [day-4:day]) | 5.0 |
| dtt_minlag | Min lag time (in seconds) | 5.0 |
| dtt_width | Width of the time lag window (in seconds) | 30.0 |
| dtt_sides | Which sides to use [both]/left/right | both |
| dtt_mincoh | Minimum coherence on dt measurement, MWCS points with values lower than that will **not** be used in the WLS | 0.7 |
| dtt_maxerr | Maximum error on dt measurement, MWCS points with values larger than that will **not** be used in the WLS | 0.1 |
| dtt_maxdt | Maximum dt values, MWCS points with values larger than that will **not** be used in the WLS | 0.1 |

Filters

| | | |
|---|---|---|
| Filter low | The lower frequency bound of the Whiten function (in Hz) | 1.00 |
| Filer high | The upper frequency bound of the Whiten function (in Hz) | 2.00 |
| MWCS low | The lower frequency bound of the linear regression done in MWCS (in Hz) | 1.02 |
| MWCS high | The upper frequency bound of the linear regression done in MWCS (in Hz) | 1.98 |
| MWCS length | Window length (in seconds) to perform MWCS | 12.00 |
| MWCS step | Step (in seconds) of the windowing procedure in MWCS | 4.00 |

Supplementary Table 1. **MSNoise parameters**

References

[1] Lecocq, T., Caudron, C. & Brenguier, F. MSNoise, a Python Package for Monitoring Seismic Velocity Changes Using Ambient Seismic Noise. *Seismol. Res. Lett.* **85**, 715–726 (2014).

[2]Sens-Schönfelder, C. & Wegler, U. Passive image interferometry and seasonal variations of seismic velocities at Merapi Volcano, Indonesia. *Geophys. Res. Lett.* **33**, (2006).

[3]Schimmel, M., Stutzmann, E. & Gallart, J. Using instantaneous phase coherence for signal extraction from ambient noise data at a local to a global scale. *Geophys. J. Int.* **184**, 494–506 (2011).

[4]Mousavi, S. M., Ellsworth, W. L., Zhu, W., Chuang, L. Y. & Beroza, G. C. Earthquake transformer—an attentive deep-learning model for simultaneous earthquake detection and phase picking. *Nat. Commun.* **11**, 3952 (2020).

[5]Herrmann, R.B., 2013. Computer programs in seismology: An evolving tool for instruction and research. *Seismological Research Letters*, *84*(6), pp.1081-1088.

[6]Terakawa, T. *et al.* Monitoring eruption activity using temporal stress changes at Mount Ontake volcano. *Nat. Commun.* **7**, 10797 (2016).

[7]Pacheco, C. and Snieder, R., 2005. Time-lapse travel time change of multiply scattered acoustic waves. *The Journal of the Acoustical Society of America*, *118*(3), pp.1300-1310.
